# Assessment of retinal microvascular changes in patients with systemic lupus erythematosus using optical coherence tomography angiography

**DOI:** 10.1186/s40942-025-00677-2

**Published:** 2025-05-08

**Authors:** Ahmed Ibrahim Basiony, Sameh Mohamed Elgouhary, Hadeer Elbasuony Mohamed, Enas Sobhy Zahran

**Affiliations:** 1 Department of Ophthalmology Faculty of Medicine, Menoufia University, Menoufia, Egypt; 2 Mansoura Ophthalmology Hospital, Mansoura, Dakahlia, Egypt; 3 Department of Internal Medicine and Rheumatology Faculty of Medicine, Menoufia University, Menoufia, Egypt; 4 Department of ophthalmology Faculty of medicine, Yasin Abdelghaffar St. Shebin Elkom, Menoufia, Egypt

**Keywords:** Eye, Lupus nephritis, Optical coherence tomographic angiography, Retina, Systemic lupus erythematosus

## Abstract

**Background:**

It is evident that the physiopathological pathways of ocular and renal microvascular tissues in patients with systemic lupus are similar. Previously, this was confirmed by employing traditional fundus examination, optical coherence tomography, and high-resolution color electroretinography. Recent years have seen the development of Optical Coherence Tomography Angiography (OCTA) as a non-intrusive procedure that can be employed to image the microvasculature of the retina and choroid.

**Objective:**

The aim of this study is to assess the correlation between renal functional and histologic features with the retinal microvasculature alterations in systemic lupus patients through OCTA analysis.

**Patients and methods:**

This case-control study enrolled thirty-six eyes from 18 lupus nephritis (LN) patients, thirty-six eyes from 18 systemic lupus erythematosus (SLE) patients, and thirty eyes from 15 healthy controls. An ophthalmological evaluation, including history, examination, and investigations, was conducted using OCTA for all participants. Prior to ocular examination and investigation, all SLE patients underwent a rheumatological evaluation, encompassing disease-related clinical and laboratory assessments. Specimen retrieval and renal biopsy examinations were also performed, categorizing them into lupus and lupus nephritis patients.

**Results:**

Regarding central foveal thickness (CFT) and parafoveal thickness (PFT), there were no significant differences compared to healthy subjects. A comparison of the foveal avascular zone area (FAZ-A) among the three groups revealed a significant increase in both patient groups compared to healthy controls. Whole superficial capillary plexus (SCP) vascular density (VD) in the parafoveal and foveal regions showed a significant reduction in both SLE patient groups compared to healthy controls (HC). Specifically, SCP values were 42.65 ± 2.23% in the SLE with nephritis group, 44.88 ± 2.09% in the SLE without nephritis group, and 49.10 ± 3.12% in the healthy control group. SCP parafoveal VD values were 40.77 ± 3.27% in SLE with nephritis, 47.19 ± 2.63% in SLE without nephritis, and 50.98 ± 4.80% in healthy controls. SCP foveal VD was 18.96 ± 3.43% in SLE with nephritis, 21.61 ± 4.00% in SLE without nephritis, and 24.16 ± 2.69% in healthy controls. The whole deep capillary plexus (DCP), parafoveal, and foveal VD were significantly reduced in the SLE with nephritis group but showed only marginal differences in the SLE without nephritis group compared to healthy controls, as DCP values were 48.04 ± 3.93% in SLE with nephritis, 53.63 ± 2.19% in SLE without nephritis, and 54.88 ± 3.57% in healthy controls. DCP parafoveal VD was 54.56 ± 2.37% in SLE with nephritis, 56.93 ± 1.90% in SLE without nephritis, and 57.39 ± 5.99% in healthy controls. DCP foveal VD was 34.42 ± 3.12% in SLE with nephritis, 41.96 ± 3.19% in SLE without nephritis, and 42.55 ± 7.74% in healthy controls.

**Conclusion:**

OCT angiography has a considerable role in the detection of the early changes of the retinal vascular plexus in patients with SLE, especially those with lupus nephritis, even before the development of retinopathy.

**Supplementary Information:**

The online version contains supplementary material available at 10.1186/s40942-025-00677-2.

## Introduction

Systemic lupus erythematosus (SLE) is an autoimmune disease that affects multiple organ systems. Lupus nephritis (LN) is one of the most common and serious organ-threatening complications of SLE, affecting up to 40% of patients [1].

Kidney biopsy remains the most reliable diagnostic method for lupus-induced nephritis [2]. The deposition of immune complexes is among the key mechanisms by which lupus nephritis damages both the glomerular and tubulointerstitial tissues [3].

In SLE, retinal vasculopathy is the second most common ocular manifestation after keratoconjunctivitis sicca. It can present with subtle changes or result in permanent visual loss [4]. Lupus retinopathy (LR) is characterized by microcirculatory disturbances such as arteriolar stenosis, cotton wool spots, and retinal hemorrhages. Severe cases may be complicated by central retinal vein or artery occlusion [5]. Notably, approximately 88% of patients with LR have active SLE, making the presence of retinopathy a potential marker of disease activity [6].

Although fundus fluorescein angiography (FFA) is commonly used to assess retinal involvement in SLE, it does not provide distinct visualization of the superficial and deep vascular plexuses [7]. In contrast, optical coherence tomography angiography (OCTA) has emerged as a valuable, noninvasive imaging tool capable of detecting preclinical microvascular changes in systemic diseases like SLE [8, 9]. OCTA allows for detailed imaging of the retinal microvasculature, measurement of retinal layer thickness, and assessment of blood flow—without the need for intravenous dye injection [10].

The aim of this study is to assess the correlation between ocular and renal microvascular changes in patients with SLE and lupus nephritis using optical coherence tomography angiography.

## Patients and methods

This case-control study was conducted at Menoufia University Hospital and included 72 eyes from 36 patients diagnosed with systemic lupus erythematosus (SLE). Of these, 36 eyes belonged to 18 patients with lupus nephritis (LN), and 36 eyes to 18 SLE patients without nephritis. Additionally, 30 eyes from 15 healthy individuals served as controls. The study was conducted in the inpatient and outpatient clinics of the departments of ophthalmology and rheumatology at the faculty of medicine.

Patients were diagnosed with SLE according to the Systemic Lupus International Collaborating Clinics (SLICC) 2012 criteria [11]. Informed consent was obtained from all participants after they were provided with sufficient information about the purpose and nature of the research. The study adhered to the ethical principles outlined in the Declaration of Helsinki and was approved by the Institutional Review Board (IRB No. 2/2021 OPHT5).

Inclusion criteria were as follows: adults aged 18–60 years diagnosed with SLE, best corrected visual acuity (BCVA) between 6/60 and 6/6, and OCTA scan signal strength index (SSI) of 6 or higher. Patients were excluded if they had a history of systemic diseases such as diabetes, hypertension, rheumatoid arthritis, systemic sclerosis, mixed connective tissue disease, or Sjögren’s syndrome. Ocular conditions including retinal vein occlusion, vitreous hemorrhage, dense cataract, drusen-like deposits, focal atrophy, retinal pigment epithelium detachment, ocular trauma, drug-induced retinopathy, or prior vitreoretinal surgery are also excluded.

All enrolled subjects underwent comprehensive ophthalmologic assessment, which included history, examination, and investigations using a database computer program that was specifically designed for data entry and analysis, following a pre-established protocol.

- Visual Acuity Assessment using the Autoref/Keratometer ARK-1 (NIDEK Co., Aichi, Japan, 2013), with results converted to decimal format (dc).
- Slit-Lamp Biomicroscopy (SL-D7, Topcon, Tokyo, Japan) for anterior segment examination.
- Intraocular Pressure Measurement using a Goldmann applanation tonometer (Shin Nippon, Japan).
- Fundus Examination using both indirect ophthalmoscopy (Model AAIO-7, Appasamy Associates, India, 2014) and slit-lamp biomicroscopy with a Volk + 90D non-contact lens.
- Rheumatological Assessment: Each patient with SLE underwent rheumatologic evaluation. The SLE Disease Activity Index 2000 (SLEDAI-2 K) was used to assess disease activity [12].
- Laboratory Investigation: Renal function was assessed using Blood Urea Nitrogen (BUN), serum creatinine levels (normal: 15–40 mg/dL), 24-hour proteinuria (PTU) measured via enzymatic colorimetric assay (normal: ≤300 mg/24 h), Antinuclear antibodies (ANA) (positive if ≥ 1:160), and anti-dsDNA. All tests and renal biopsy results were already done to all study cases by internal medicine specialists before ocular examination and OCTA imaging and documented in patients’ files, categorizing them into lupus and lupus nephritis patients.

### Acquisition of optical coherence tomography angiography

OCTA scans were acquired using the RTVue-XR Avanti spectral domain system (Optovue Inc., Fremont, CA, USA; software version 2017.1.0.151). A 6-mm macular-centered scan was obtained for each eye, focusing on the fovea (6 × 6 mm scan was used). The device automatically segmented the superficial (SCP) and deep capillary plexuses (DCP), with manual corrections applied as needed for minor segmentation errors using B-scans. Manual segmentation allows the user to retrace the mislabeled retinal layer. This action generates larger or smaller slabs to better assess pathology of interest. Scans with fixation instability (SSI < 5), significant motion artifacts, or major segmentation errors were excluded. The foveal avascular zone (FAZ) area, as well as foveal and parafoveal retinal thickness, were automatically measured using the device’s built-in software.

### Statistical analysis

For the quantitative processing and analysis of pre-coded data, the Statistical Package for the Social Sciences (SPSS), version 25 (IBM Corp., Armonk, NY, USA), was utilized. Descriptive statistics for numerical variables were expressed as means and standard deviations. The Kolmogorov–Smirnov test was used to assess the normality of data distribution. Qualitative variables were presented as frequencies and percentages. Comparisons between qualitative variables were conducted using the chi-square (χ²) test. For comparisons between quantitative variables, independent samples t-tests were used when the data followed a normal distribution. For variables that did not meet the assumptions of normality, non-parametric tests such as the Kruskal–Wallis test and the Mann–Whitney U test were employed. Pearson’s correlation coefficient was used to assess the strength and direction of correlations between continuous variables.

## Results

In the current study, seventy-two eyes of 36 patients diagnosed with SLE, thirty-six eyes of 18 patients with SLE-LN, thirty-six eyes of 18 patients with SLE without kidney involvement, and thirty eyes of 15 healthy controls (HC) were evaluated. The study included 11 males (21.6%) and 40 females (78.4%). The mean ages were 33.94 ± 9.95, 34.39 ± 9.39, and 32.93 ± 6.75 years in the three groups, respectively. The mean disease duration was 6.67 ± 4.75 years in the SLE-LN group and 4.84 ± 3.11 years in the SLE group. Regarding BCVA, there was no significant difference between the two patient groups; however, BCVA was lower in both patient groups compared to healthy controls, though the difference was not statistically significant (p1 = 0.12, p2 = 0.064). (Table 1)

**Table 1:** Distribution of the studied patients according to demographic data

Variables	Group (1) (SLE with Nephritis)	Group (2) (SLE without Nephritis)	Group (3) Controls
Gender			
Males (*n* = 11)	3 (16.7%)	3 (16.7%)	5 (33.3%)
Females (*n* = 40)	15 (83.3%)	15 (83.3%)	10 (66.7%)
Total (*n* = 51)	18 (100%)	18 (100%)	15 (100%)
Positive Family History	6 (33.3%)	4 (22.2%)	—
Age (years)			
Range	18–53	19–50	22–46
Mean ± SD	33.94 ± 9.95	34.39 ± 9.36	32.93 ± 6.75
Statistical Significance			t = 0.0173, *p* = 0.892 (NS)
Disease Duration (years)	6.67 ± 4.75	4.84 ± 3.11	—
BCVA (decimal)			
Mean ± SD	0.57 ± 0.17	0.68 ± 0.20	0.84 ± 0.18
BCVA p-values			
P1 (Group 1 vs. Group 2)	0.12		
P2 (Group 1 vs. Group 3)	0.064		
P3 (Group 2 vs. Group 3)	0.113		

FH: Family History; BCVA: Best Corrected Visual Acuity; SLE: Systemic Lupus Erythematosus; NS: Non-significant

Table [1]: A total of 36 SLE patients were divided into two groups: Group (1) included 18 patients with lupus nephritis, and Group (2) included 18 patients without nephritis. Group (3) consisted of 15 age- and sex-matched healthy individuals as controls. The sample included 11 males (21.6%) and 40 females (78.4%) across all groups. The mean ages were 33.94 ± 9.95, 34.39 ± 9.36, and 32.93 ± 6.75 years for Groups 1, 2, and 3, respectively, with no statistically significant difference between them (*p* = 0.892)

The mean disease duration was longer in Group (1) than in Group (2). Regarding BCVA, although visual acuity appeared lower in Group (1) compared to Groups (2) and (3), and Group (2) lower than Group (3), the differences did not reach statistical significance (*p* > 0.05)

Both SLE groups were similar in clinical signs, symptoms, and in some laboratory findings, as all patients are positive for ANA and anti-dsDNA, but regarding BUN and creatinine levels were elevated in SLN patients compared to SL patients without nephritis. The Systemic Lupus Erythematosus Disease Activity Index (SLEDAI-2 K) was significantly higher in the SLE-LN group compared to the SLE group without nephritis. The mean SLEDAI-2 K score was 8.44 ± 3.4 in the SLE-LN group and 4.0 ± 0.0 in the SLE group, indicating moderate disease activity. (Table 2)

**Table 2:** Signs and symptoms of the patients’ groups

Signs and Symptoms	Group 1 (*n* = 18) No.	Group 1%	Group 2 (*n* = 18) No.	Group 2%	Test of Significance (χ² / t, *p*)
**Malar rash**	16	88.9	15	83.3	χ² = 0.742 *p* = 0.135
**Arthritis**	5	27.8	5	27.8	χ² = 0.000 *p* = 1.000
**Serositis**	3	16.7	2	11.1	χ² = 1.368 *p* = 0.058
**Oral ulcers**	18	100	18	100	χ² = 0.000 *p* = 1.000
**ANA titer**	18	100	18	100	χ² = 0.000 *p* = 1.000
**dsDNA antibody titer**	18	100	18	100	χ² = 0.000 *p* = 1.000
**Positive LA**	4	22.2	4	22.2	χ² = 0.000 *p* = 1.000
**Positive ACL**	4	22.2	4	22.2	χ² = 0.000 *p* = 1.000
**SLEDAI Range**		2–12		4	
**SLEDAI Mean ± SD**		8.44 ± 3.4		4.0 ± 0.0	t = 5.937

Antinuclear Antibody (ANA), Double-Stranded DNA (dsDNA), Lupus Anticoagulant (LA), Anticardiolipin Antibody (ACL), Systemic Lupus Erythematosus Disease Activity Index (SLEDAI)

Table [2]: Both groups showed similar clinical signs and symptoms, with no statistically significant differences observed (*p* > 0.05). However, the SLE Disease Activity Index (SLEDAI) was significantly higher in Group 1 (patients with nephritis) compared to Group 2 (patients without nephritis), with a p-value of 0.001

**Fig. 1:**
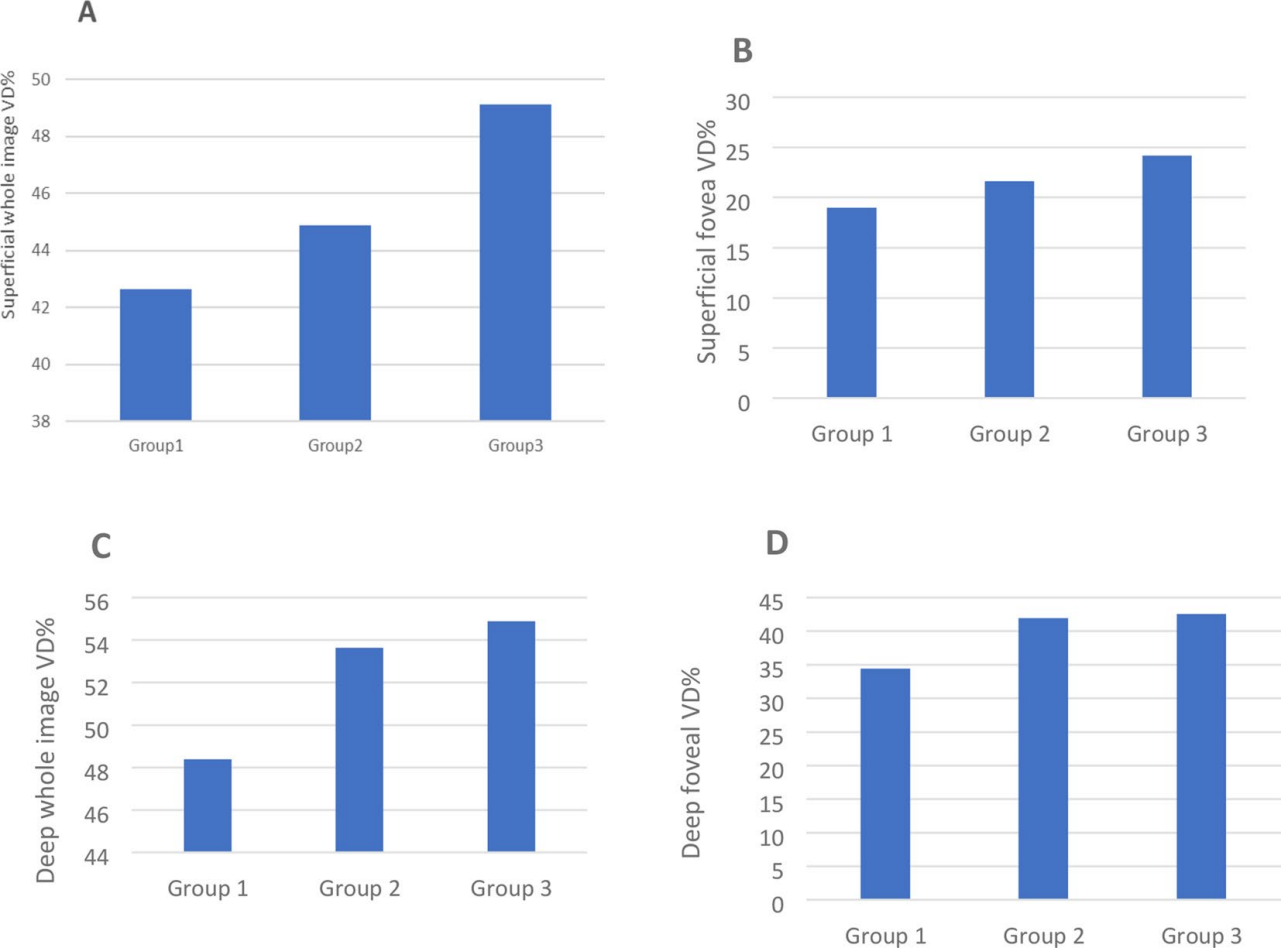
Significantly lower superficial whole (**A**) and foveal retinal vessel densities were observed in systemic lupus patients (Group 1) and systemic lupus patients without nephritis (Group 2) compared to healthy subjects (Group 3). Regarding the superficial capillary plexus (SCP), p1 = 0.019, p2 = 0.028, and p3 = 0.035. For superficial foveal vessel density, p1 = 0.028, p2 = 0.035, and p3 = 0.036. Additionally, significantly lower deep whole (**C**) and deep foveal retinal vessel densities were observed in patients with systemic lupus nephritis (Group 1) compared to healthy subjects (Group 3). However, no significant differences were found between systemic lupus patients without nephritis (Group 2) and healthy subjects (Group 3) in these parameters. Regarding the deep capillary plexus (DCP), p1 = 0.009, p2 = 0.192, and p3 = 0.026. For deep foveal vessel density, p1 = 0.007, p2 = 0.331, and p3 = 0.024

**Fig. 2:**
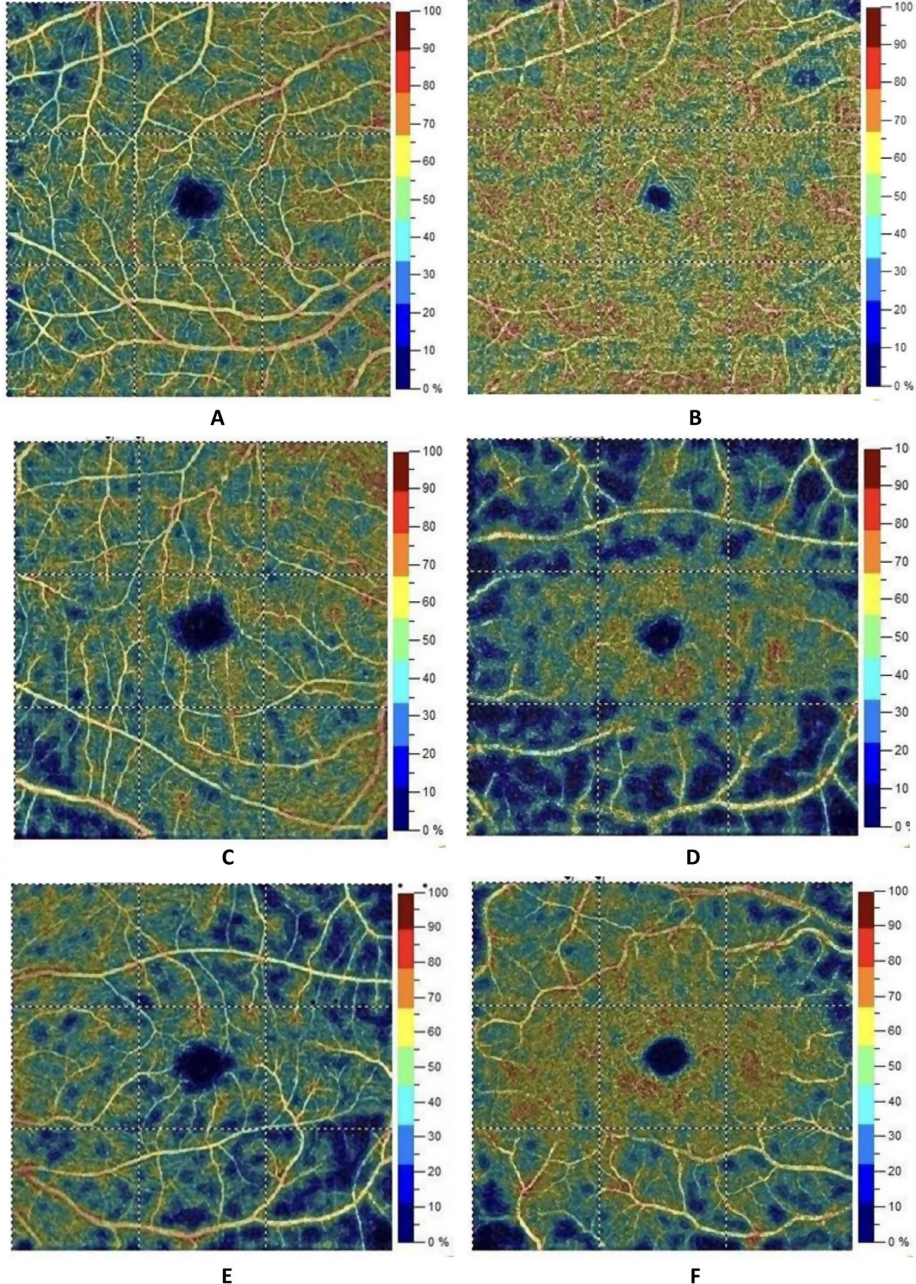
Grid-based vessel density maps of the retinal capillary plexus obtained from the Optovue AngioVue OCTA system. (**a**) Superficial retinal vessel density map and (**b**) deep retinal vessel density map of a normal subject. (**c**) and (**d**) represent the corresponding maps from an SLE patient with nephritis, while (**e**) and (**f**) represent those from an SLE patient without nephritis. The dotted lines divide the maps into nine quadrants, with vessel density percentages calculated for each quadrant. Hot colors (red, orange, and yellow) indicate areas of high vessel density, whereas cold colors (green and blue) represent areas of lower vessel density

By comparing SLE patients to healthy controls (HC) using OCTA parameters—including whole superficial capillary plexus (SCP), deep capillary plexus (DCP), superficial foveal density, and deep foveal density—the following observations were made:

A significant reduction in the density of the superficial retinal capillary plexus was found in both groups of SLE patients compared to healthy controls. The mean SCP density was 42.65 ± 2.23 in the SLE-LN group and 44.88 ± 2.09 in the SLE without nephritis group, while it was 49.01 ± 3.12 in healthy controls. The statistical significance of these differences was demonstrated by the following p-values: P1 = 0.019 (SLE-LN vs. HC), P2 = 0.028 (SLE without nephritis vs. HC), and P3 = 0.035 (SLE-LN vs. SLE without nephritis). These results indicate a significant reduction in SCP density in both SLE groups, with a more pronounced decrease in the SLE-LN group (Fig. 1A). Additionally, a significant reduction in superficial foveal density was noted in both SLE groups compared to healthy controls. The mean values were 18.96 ± 3.43 in SLE-LN, 21.61 ± 4.00 in SLE without nephritis, and 24.16 ± 2.69 in healthy controls. Corresponding p-values were: P1 = 0.028, P2 = 0.035, and P3 = 0.036, indicating a statistically significant difference with greater reduction observed in the SLE-LN group (Figs. 1B and 2a, c and e).

According to the measurements of whole deep retinal vessel density and foveal density, our findings revealed a statistically significant decrease in both parameters in the SLE-LN group compared to healthy controls (HC). The mean deep vessel and foveal densities in the SLE-LN group were 48.04 ± 3.93 and 34.42 ± 3.12, respectively, while in the HC group they were 54.88 ± 3.57 and 42.55 ± 7.74, respectively. The differences were statistically significant, with P1 values of 0.009 for deep vessel density and 0.007 for deep foveal density.

In contrast, SLE patients without nephritis showed no significant differences in these parameters compared to HC. The mean deep vessel and foveal densities were 53.63 ± 2.19 and 41.96 ± 3.19, respectively, compared to 54.88 ± 3.57 and 42.55 ± 7.74 in HC (P2 = 0.192 and 0.331, respectively). A significant decrease between the two patient groups was observed in favor of SLE-LN, with P3 values of 0.026 for deep vessel density and 0.024 for deep foveal density (Figs. 1C, amp and D and 2b, d and f).

Assessment of parafoveal density data revealed a substantial reduction in the superficial capillary plexus within the SLE group compared to HC. The mean parafoveal density was 40.77 ± 3.27 in the SLE-LN group (P1 = 0.006), and 47.19 ± 2.72 in the SLE group without nephritis (P2 = 0.03), compared to 52.22 ± 3.43 in the HC group. A notable decrease was also observed in the deep parafoveal plexus in the SLE-LN group compared to HC (54.56 ± 2.37 vs. 57.39 ± 5.66, P1 = 0.041), while no meaningful distinction was detected in the SLE group without nephritis compared to HC (56.93 ± 1.90 vs. 57.39 ± 5.66, P2 = 0.232). Significant differences between the two SLE patient groups were found in both superficial and deep parafoveal densities, favoring the SLE-LN group (P3 = 0.032 and 0.048, respectively) (Supplementary Fig. S1 A–B).

**Fig. 3:**
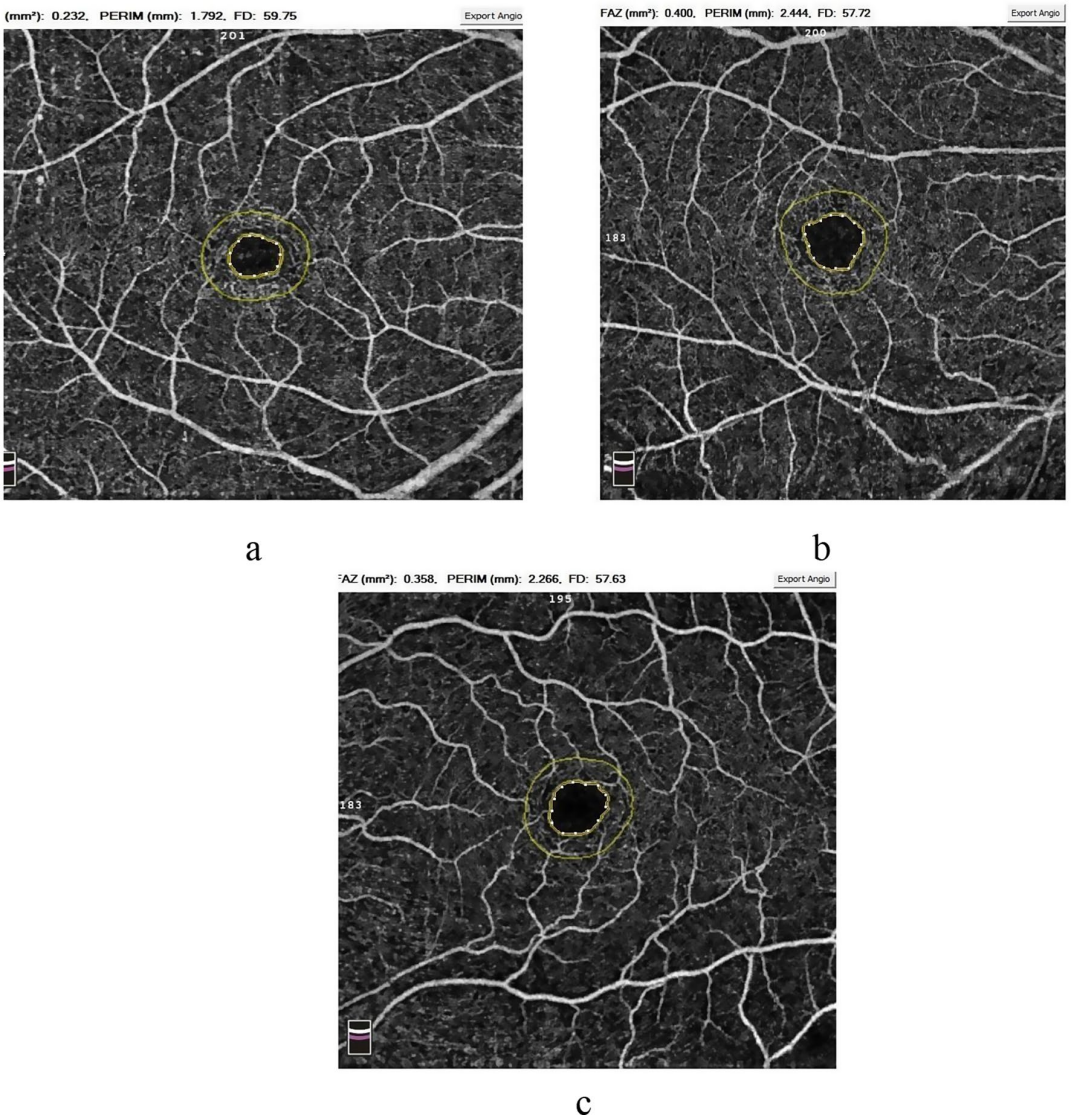
OCTA delineation of the foveal avascular zone (FAZ) area (inner yellow circle) and the FD-300 area (the region between the inner and outer yellow circles) in: (**a**) a normal subject,) **b**) an SLE patient with nephritis, and (**c**) an SLE patient without nephritis. A noticeable enlargement of the FAZ area is observed in SLE patients compared to the normal subject, indicating a significant increase in FAZ in association with disease presence

Comparative analysis of foveal avascular zone (FAZ) data showed that both SLE-LN and SLE patients had significantly larger FAZ areas compared to HC. The mean FAZ area was 0.282 ± 0.07 in the SLE-LN group, 0.234 ± 0.07 in the SLE group without nephritis, and 0.216 ± 0.03 in HC, with P1 = 0.029, P2 = 0.015, and P3 = 0.049 (Supplementary Fig. S1C; Fig. 3a, b, c). Central foveal thickness, FD300, and parafoveal thickness were also evaluated using OCTA, showing no significant differences between SLE patients and healthy controls (Table 3).

**Table 3:** Foveal and parafoveal parameters of the three studied groups

Item	Group (1) Mean ± SD	Group (2) Mean ± SD	Group (3) Mean ± SD	P1	P2	P3
**CFT** (**µm**)	238.0 ± 7.11	238.9 ± 10.7	245.2 ± 15.18	0.993	0.755	0.752
**FAZ** (**mm²)**	0.282 ± 0.07	0.234 ± 0.07	0.216 ± 0.03	0.029*	0.015*	0.049*
**FD300 (%)**	52.16 ± 4.21	53.19 ± 1.98	57.28 ± 3.84	0.263	0.295	0.297
**PFT (µm)**	314.89 ± 7.61	315.3 ± 11.3	321.68 ± 16.9	0.957	0.789	0.788

Central Foveal Thickness (CFT), Foveal Avascular Zone (FAZ), Vascular Density within 300 μm around the FAZ (FD300), Parafoveal Thickness (PFT)

Table [3]: The mean values of CFT, FAZ, and PFT were lower in Group (1) compared to Groups (2) and (3), and also lower in Group (2) compared to the control Group (3); however, these differences were not statistically significant (*p* > 0.05). Notably, FAZ was significantly larger in the SLE group with nephritis (Group 1) compared to both the non-nephritis SLE group (Group 2) and the control group (Group 3). FAZ was also significantly larger in Group (2) than in the control group (*p* < 0.05)

**Fig. 4:**
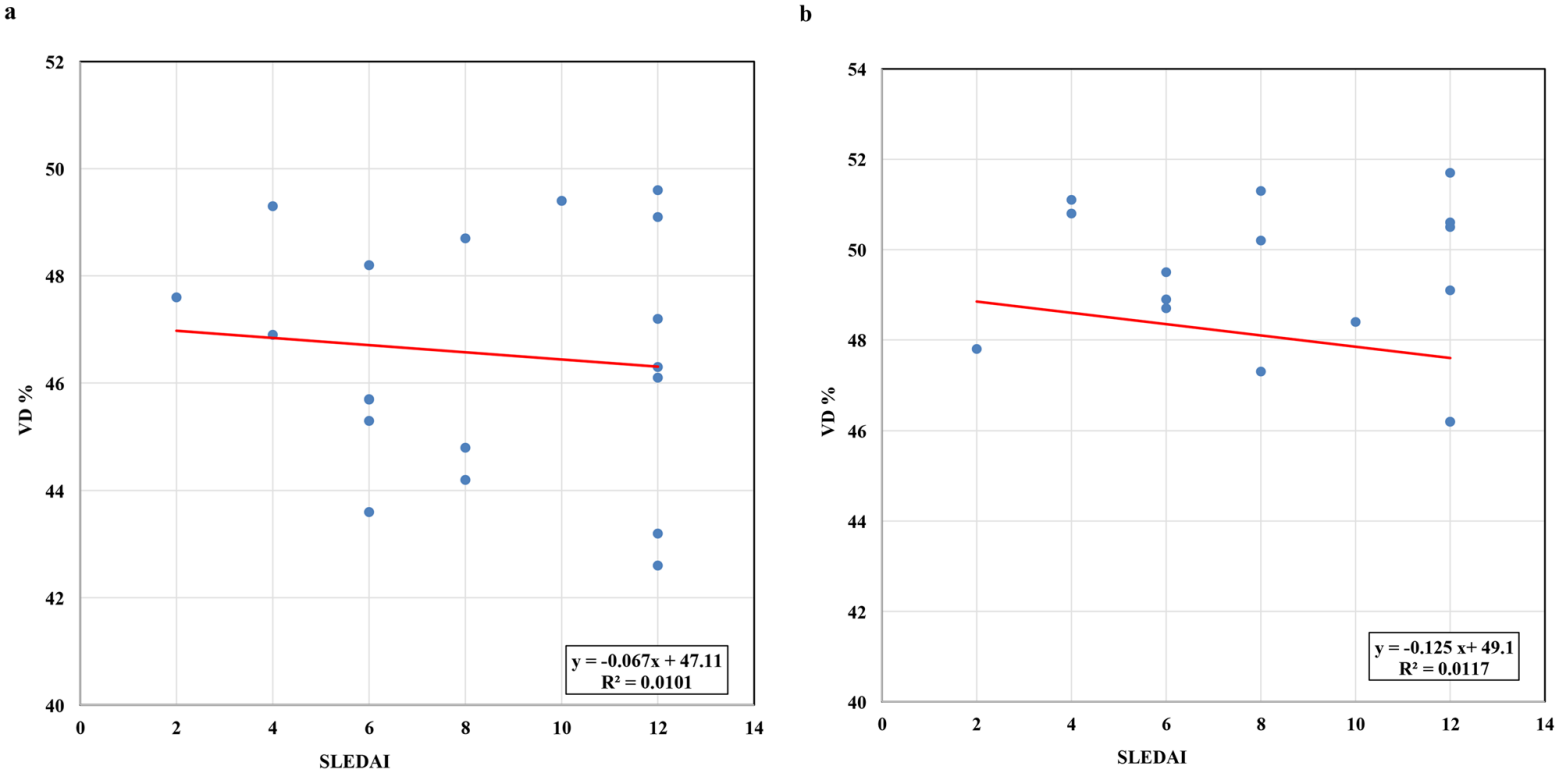
Correlation coefficient (r) between Systemic Lupus Erythematosus. Disease Activity Index and whole retinal superficial vascular density in SLE-positive nephritis patients (**a**) and whole retinal deep vascular density (**b**). There is an insignificant negative correlation between the two parameters: (*r* = -0.1005, *p* = 0.065) for the superficial vascular density and (*r* = -0.1082, *p* = 0.062) for the deep vascular density

**Fig. 5:**
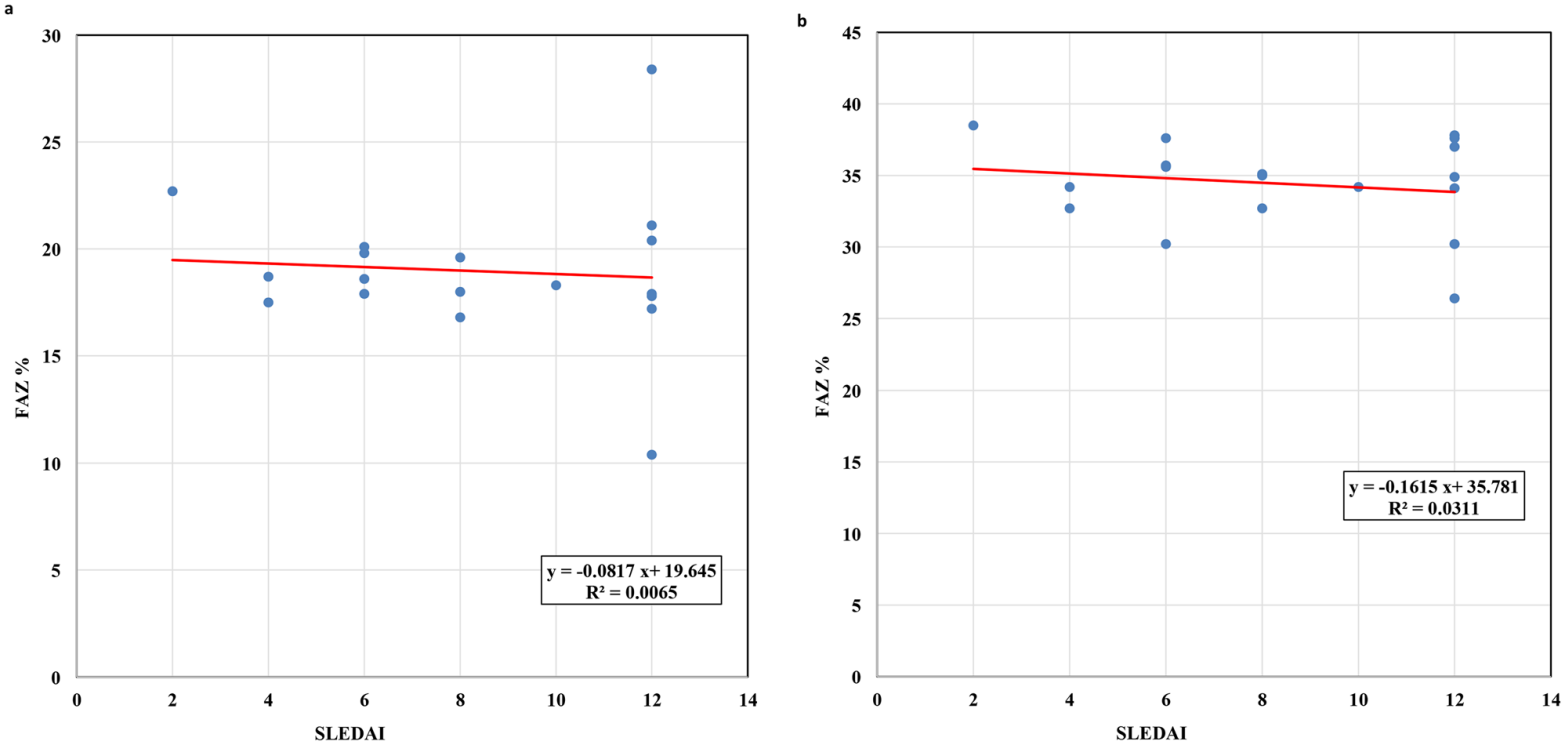
Correlation coefficient (r) between Systemic Lupus Erythematosus. Disease Activity Index and superficial foveal avascular zone in SLE-positive nephritis patients (**a**) and deep foveal avascular zone density (**b**). There is an insignificant negative correlation between the two parameters: (*r* = -0.0806, *p* = 0.062) for the superficial foveal avascular zone and (*r* = -0.1764, *p* = 0.059) for the deep foveal avascular zone density

Regarding disease activity, a negative correlation existed between the SLEDAI-2 K score and both superficial and deep whole retinal vessel densities (*r* = -0.1005, *p* = 0.065; *r* = -0.1082, *p* = 0.062, respectively) (Fig. 4a, b). Similarly, negative correlations were observed between the SLEDAI-2 K score and both superficial and deep foveal densities (*r* = -0.0806, *p* = 0.062; *r* = -0.1764, *p* = 0.059, respectively) (Fig. 5a, b). These findings suggest that as disease activity increases, there is a trend toward decreased superficial and deep vascular densities, although the correlations did not reach statistical significance.

## Discussion

Systemic lupus erythematosus (SLE) is a chronic, multi-system autoimmune disease that can obviously affect various parts of the eye. Microvascular changes in SLE can contribute significantly to disease-related morbidity and even mortality. Retinal microvascular involvement may reflect systemic vascular damage in other organs. Currently, one of the most advanced techniques used to assess the morphological changes in retinal microvasculature is optical coherence tomography angiography (OCTA) [13]. Therefore, our case-control study was conducted using OCTA to identify potential biomarkers that could predict early anatomical changes associated with SLE. OCTA images were taken from 72 eyes of 36 patients diagnosed with SLE, categorizing them into 36 eyes from patients with lupus nephritis and 36 eyes from patients without nephritis based on laboratory and pathological classification of internal medicine specialists. Additionally, 30 eyes from healthy individuals were included as a control group.

The participants’ ages ranged from 18 to 55 years. Among them, 12 were males (16.6%) and 60 were females (83.3%), resulting in a female-to-male ratio of 5:1, which reflects the higher prevalence of the disease among females. This finding is consistent with Pichi et al. (2020), who included thirty eyes of 15 SLE patients in their study, reporting that 9 patients were female (60%) and 6 were male (40%), confirming the female predominance of SLE [14]. Nusbaum et al. (2020) also reported that SLE is nearly nine times more prevalent in women than in men throughout the lifespan [15].

In our study, the mean best-corrected visual acuity (BCVA) was 0.57 ± 0.17 in SLE patients with nephritis, 0.68 ± 0.20 in SLE patients without nephritis, and 0.84 ± 0.18 in healthy controls. These findings are in agreement with those of Arfeen et al. (2020), whose research found that BCVA was marginally lower in the SLE group compared to the control group after analyzing 20 eyes from SLE patients and 20 eyes from healthy subjects [16]. This decline may be linked to a marked reduction in the vascular density of the SLE group. However, the interrelation between visual acuity and retinal vessel density remains perplexing and requires further investigation [17, 18].

In contrast to our results, Sultan et al. (2019) confirmed a correlation between SLE and visual acuity, primarily due to capillary perfusion loss, especially in the deep capillary plexus [19]. In the same way, Mimier-Janczak et al. (2022), who studied 57 eyes from SLE patients and 56 eyes from healthy individuals, reported a significantly lower visual acuity (*p* = 0.019) in the SLE group. This decline may be attributed to varying degrees of capillary loss, though the strength of this correlation remains unclear due to a lack of comprehensive data [13].

Regarding central foveal and parafoveal thickness, no statistically significant differences were found between SLE patients and healthy controls in our study. This finding aligns with Arfeen et al. (2020), who also reported no difference in central foveal thickness (CFT) between SLE patients and healthy individuals [16]. Furthermore, Işık et al. (2021) observed no significant differences in CFT between the SLE and control groups [20]. In contrast, Conigliaro et al. (2022), who analyzed 46 eyes of 23 lupus nephritis (LN) patients, 32 eyes of 16 SLE patients without nephritis, and 42 eyes of 21 healthy controls, demonstrated a significantly thinner foveal avascular zone (FAZ) in both SLE groups (*p* < 0.0001 in SLE-LN and *p* = 0.003 in SLE without nephritis) compared to healthy controls. Additionally, the parafoveal area was notably thinner in SLE patients than in healthy subjects [21].

Regarding the foveal avascular zone (FAZ), our study observed a significant increase in FAZ area in both patient groups compared to healthy subjects, with a more pronounced enlargement in the SLE-LN group. Similarly, Forte et al. (2019), in a study involving 10 SLE patients on hydroxychloroquine (HCQ) and 18 healthy controls, revealed an increased FAZ size across all three capillary plexuses [22]. Also, Pichi et al. (2020) observed FAZ enlargement in their study of 15 SLE patients without ophthalmological manifestations (mean FAZ area: 0.22 ± 0.12 mm²), attributing this change to retinal vessel hypoperfusion and tissue hypoxia observed in patients with subclinical SLE [14].

In the same way, Mihailovic et al. (2020), in a study of 19 eyes from SLE patients without HCQ retinopathy and 19 eyes from healthy individuals, found that the FAZ area in the SLE group was significantly larger than in the control group (0.279 ± 0.085 mm² vs. 0.218 ± 0.067 mm²; *p* = 0.019) [23]. On the contrary, Arfeen et al. (2020) and Işık et al. (2021) showed no significant differences in FAZ area between SLE patients and healthy controls [16, 20].

According to vascular density changes, our study demonstrated a significant decrease in superficial whole, parafoveal, and foveal densities in SLE patients compared to healthy subjects, with a more pronounced reduction observed in patients with systemic lupus nephritis (SLN) than in those without nephritis. Similar findings were reported by Conigliaro et al. (2019), who conducted a study on 52 eyes of 26 SLE patients and observed a significant reduction in superficial whole en face, parafoveal, and foveal vessel densities [8]. Likewise, Bao et al. (2020), who included 32 SLE patients (58 eyes) without lupus retinopathy (NLR), 14 patients (22 eyes) with lupus retinopathy (LR), and 50 healthy controls (50 eyes), concluded that superficial retinal capillary plexus (SRCP) density in SLE patients without LR was substantially lower compared to healthy controls in almost all regions and further decreased in those with LR (*P* < 0.05) [24]. Additionally, Işık et al. (2021) reported reduced inner, outer, and full vessel density and perfusion density in SLE patients [20].

Our results regarding differences between patients with and without lupus nephritis align with those of Conigliaro et al. (2022), who revealed a significant reduction in superficial whole en face vessel density in both SLE groups compared to healthy controls, with a more marked reduction in the LN group. This study also supported our results regarding superficial foveal density, as OCTA evaluation showed a significant reduction in superficial foveal vessel density in SLE patients with LN compared to those without kidney involvement. Furthermore, parafoveal density was significantly reduced in the superficial plexus of the SLE group compared to healthy controls [21]. Pichi et al. (2020) and Mihailovic et al. (2020) also reported reduced vessel density in the en face superficial capillary plexus among SLE patients [14, 23].

Regarding the assessment of deep retinal vessel density, our study reported significant differences between the two patient groups. There was a marked reduction in deep whole, foveal, and parafoveal densities in the SLN group compared to healthy controls, whereas no significant difference was found in SLE patients without kidney involvement. In high agreement with Conigliaro et al. (2022), who reported a significant reduction in deep whole en face and deep foveal vessel density in SLN patients compared to healthy controls, while SLE patients without kidney involvement didn’t differ significantly in deep density compared to HC [21]. However, they reported no significant differences in the deep parafoveal plexus between systemic lupus patients and healthy controls. This contrasts slightly with our findings, which revealed a significant decrease in deep parafoveal density in the SLN group compared to healthy controls, although this reduction was not statistically significant in the SLE group without nephritis [21].

On a broader scale, we reviewed other studies regarding the impact of OCTA on the various rheumatologic diseases. Concerning Rheumatoid arthritis (RA) Lee, H.Yet al.(2023), found that there was a significant decrease in both SVD and DVD in the RH group compared with the control group [25]. Similarly, Ayar, K et al. (2021) found that macular retinal capillary perfusion density (CPD) was lower in RA patients compared to HCs [26]. In addition, Pierluigi Iacono et al. (2022), who observed patients with recent diagnoses of “definite RA,” mentioned that OCTA data analysis revealed a statistically significant reduction in superficial vessel density (VD) in RA patients; however, these differences did not reach statistical significance [27].

Regarding primary Sjögren’s syndrome (PSS), Neslihan Parmak Yener et al. (2022) revealed that retinal capillary density (CD) in the deep retinal capillary plexus was lower in PSS patients compared to healthy individuals [28]. Also, Ren Liu et al. (2022) noticed a significantly lower superficial vessel density (SVD) in SS patients than in controls (*p* < 0.05) [29].

Concerning systemic sclerosis, Rosario Foti et al. (2024) compared a group of 32 systemic sclerosis patients with 9 healthy controls. The study revealed a statistically significant reduction in vessel density (VD) of the entire deep capillary plexus (DCP) and VD of both the superficial (SCP) and deep capillary plexuses within the ETDRS grid in the patient group compared to controls (*p* < 0.001) [30].

The previous studies, which encircle a range of rheumatologic diseases and their assessment using OCTA, highlight the sigificance of OCTA in the early diagnosis of these diseases and, consequently, in their treatment. Therefore, incorporating OCTA into routine evaluation of SLE patients offers valuable insights that could lead to more personalized and appropriate management.

In our study, the SLEDAI score—which reflects disease activity—showed an insignificant negative correlation with both superficial and deep whole retinal and foveal vessel densities. This suggests that as disease activity increases, there is a tendency toward reduced vessel densities. Similarly, Conigliaro et al. (2019) found an inverse association between SLEDAI scores and retinal vessel density in SLE patients [8]. In support of this, Ermurat and Koyuncu (2022) published a study involving OCTA findings in SLE patients with varying levels of disease activity, including 47 eyes of SLE patients and 41 eyes of healthy controls. Their results concluded a significant reduction in superficial capillary plexus vessel density in patients with high disease activity—particularly in the inferior hemi-retina, fovea, temporal parafovea, and inferior and temporal perifoveal areas. Additionally, deep capillary plexus vessel density was significantly reduced in all foveal and parafoveal regions [31].

Oppositely, other studies found no correlation between SLEDAI scores and retinal vessel density [16, 32]. Jesus D et al. further explained that the SLEDAI score may not be a fully reliable indicator of a patient’s condition due to its subjective nature, complexity, and low sensitivity to subtle clinical changes. These limitations may affect its accuracy in evaluating SLE disease activity [33].

## Limitations

Our study has several limitations that should be acknowledged. Firstly, the small sample size of patients may limit the generalizability of the findings. Secondly, the cross-sectional design of the study restricts the ability to follow up the study cases. Lastly, the drug-induced toxicity of the associated therapy of the patients has to be considered in the future research, which may have overlapping consequences. These limitations should be carefully taken into consideration in subsequent studies to enhance the robustness and validity of the findings.

## Conclusions

OCTA play a considerable role in detecting early changes in the retinal vascular plexus, even before the appearance of manifest symptoms in patients with SLE. Notably, the alterations in both superficial and deep retinal vascular density, as well as the enlargement of the FAZ area, were more pronounced in SLN patients compared to those with SLE without kidney involvement. Therefore, patients with lupus nephritis appear to be more susceptible to ocular involvement and warrant closer monitoring. Meanwhile, these findings highlight the merit of OCTA in allowing early intervention and modification of treatment strategy, including precise initiation of immunosuppressive therapy, potentially preventing further retinal damage and preserving vision.

## Electronic supplementary material

Below is the link to the electronic supplementary material.

Supplementary Material 1

## Data Availability

The data sets used and/or analyzed during the current study are available from the corresponding author on reasonable request.

## References

[1] Fanouriakis A, Kostopoulou M, Chee MAK, et al. 2019 Update of the joint European league against rheumatism and European renal association–European Dialysis and transplant association (EULAR/ERA EDTA) recommendations for the management of lupus nephritis. Ann Rheum Dis. 2020;79:713–23. 32220834 10.1136/annrheumdis-2020-216924

[2] Hahn BH, Mcmahon MA, Wilkinson A, et al. American college of rheumatology guidelines for screening, treatment, and management of lupus nephritis. Arthritis Care Res (Hoboken). 2012;64:797–808. 22556106 10.1002/acr.21664 PMC3437757

[3] Maciej L, Anders H-J. The pathogenesis of lupus nephritis. JASN. 2013;24:1357–66. 23929771 10.1681/ASN.2013010026 PMC3752952

[4] Kharel Sitaula R, Shah DN, Singh D. Role of lupus retinopathy in systemic lupus erythematosus. J Ophthalmic Inflamm Infect. 2016;6:15. 27174124 10.1186/s12348-016-0081-4 PMC4864796

[5] Mizuno Y, Nishide M, Wakabayashi T, Nishida K, Kumanogoh A. OCTA, a sensitive screening for asymptomatic retinopathy, raises alarm over systemic involvements in patients with SLE. Rheumatology. 2020;79:e17. 10.1136/annrheumdis-2018-214751. 10.1136/annrheumdis-2018-214751 30487146

[6] Silpa-Archa S, Lee JJ, Foster CS. Ocular manifestations in systemic lupus erythemato sus. Br J Ophthalmol. 2016;100:135–41. 25904124 10.1136/bjophthalmol-2015-306629

[7] Chalam KV, Sambhav K, Ophthalmic Vis J. Res. 2016 Jan-Mar;11(1):84–92. 10.4103/2008-322X.180709. PMID: 27195091; PMCID: PMC4860994. 10.4103/2008-322X.180709 PMC4860994 27195091

[8] Conigliaro P, Cesareo M, Chimenti MS, Triggianese P, Canofari C, Aloe G, Nucci C, Perricone R. Evaluation of retinal microvascular density in patients affected by systemic lupus erythematosus: an optical coherence tomography angiography study. Ann Rheum Dis. 2019;78(2):287–9. 10.1136/annrheumdis-2018-214235. Epub 2018 Sep 21. PMID: 30242032. 30242032 10.1136/annrheumdis-2018-214235

[9] Triggianese P, Cesareo M, Guarino MD, et al. Evaluation of retinal microvascular per fusion in hereditary angioedema: a case-con trol study. Orphanet J Rare Dis. 2020;15:20. 31952522 10.1186/s13023-019-1263-6 PMC6969431

[10] Kashani AH, Chen CL, Gahm JK, et al. Optical coherence tomography angiography: A comprehensive review of current methods and clinical applications. Prog Retin Eye Res. 2017;60:66–100. 28760677 10.1016/j.preteyeres.2017.07.002 PMC5600872

[11] Petri M, Orbai A, Alarco GS, et al. Derivation and validation of the systemic lupus international collaborating clinics classifica- Tion criteria for systemic lupus erythematosus. Arthritis Rheum. 2012;64:2677–86. 10.1002/art.34473. 22553077 10.1002/art.34473 PMC3409311

[12] Gladman DD, Ibañez D, Urowitz MB, et al. Systemic lupus erythematosus disease activity index 2000. J Rheumatol. 2002;29:288–91. 11838846

[13] Mimier-Janczak M, Kaczmarek D, Proc K, Misiuk-Hojło M. Evaluation of subclinical retinal disease in patients affected by systemic lupus erythematosus with no evidence of ocular Involvement—An optical coherence tomography angiography original study. J Clin Med. 2022;11(24):7417. 36556032 10.3390/jcm11247417 PMC9780932

[14] Pichi F, Woodstock E, Hay S, Neri P. Optical coherence tomography angiography findings in systemic lupus erythematosus patients with no ocular disease. Int Ophthalmol. 2020;40:2111–8. 32333338 10.1007/s10792-020-01388-3

[15] Nusbaum JS, Mirza I, Shum J, Freilich RW, Cohen RE, Pillinger MH et al. Sex differences in systemic lupus erythematosus: epidemiology, clinical considerations, and disease pathogenesis. In Mayo Clinic Proceedings: 2020; (Vol. 95, No. 2, pp. 384–394). Elsevier.). 10.1016/j.mayocp.2019.09.012 32029091

[16] Arfeen SA, Bahgat N, Adel N, Eissa M, Khafagy MM. Assessment of superficial and deep retinal vessel density in systemic lupus erythematosus patients using optical coherence tomography angiography. Graefe Arch Clin Exp Ophthalmol. 2020;258:1261–8. 10.1007/s00417-020-04626-7 32162113

[17] Hwang TS, Gao SS, Liu l. Automated quantification of capillary nonperfusion using optical coherence tomography angiography in diabetic retinopathy. JAMA Ophthalmol. 2016;134:367–73. 26795548 10.1001/jamaophthalmol.2015.5658 PMC4978127

[18] Samara WA, Shahlaee A, Adam MK. Quantification of diabetic macular ischemia using optical coherence tomography angiography and its relationship with visual acuity. Ophthalmology, 2016:1–10. 10.1016/j.ophtha.2016.10.008 27887743

[19] Sultan W, Asanad S, Karanjia R, Sadun AA. Long-term Attenuation ofthe deep capillary plexus in SLE utilizing OCTA. Can J Ophthalmol Can D’ophtalmologie. 2019: 1–5. 10.1016/j.jcjo.2018.10.013 31358173

[20] Işık MU, Akmaz B, Akay F, Güven YZ, Solmaz D, Gercik Ö, et al. Evaluation of subclinical retinopathy and angiopathy with OCT and OCTA in patients with systemic lupus erythematosus. Int Ophthalmol. 2021;41:143–50. 32851556 10.1007/s10792-020-01561-8

[21] Conigliaro P, Giannini C, Ferrigno S, Nesi C, Fonti GL, Chimenti MS, Triggianese P, Aiello F, Nucci C, Bergamini A, Cesareo M. Assessment of microvascular involvement in lupus nephritis patients by retinal octangiography and kidney biopsies. Clin Exp Rheumatol. 2023;41(3):581588. 10.55563/clinexprheumatol/p1q482. Epub 2022 Jul 28. PMID: 35916306. 10.55563/clinexprheumatol/p1q482 35916306

[22] Forte R, Haulani H, Dyrda A, Jürgens I. Swept source optical coherence tomography angiography in patients treated with hydroxychloroquine: correlation with morphological and functional tests. Br J Ophthalmol. 2021;105(9):1297–301. 10.1136/bjophthalmol-2018-313679. Epub 2019 Mar 6. PMID: 30842084. 30842084 10.1136/bjophthalmol-2018-313679

[23] Mihailovic N, Leclaire MD, Eter N, Brücher VC. Altered microvascular density in patients with systemic lupus erythematosus treated with Hydroxychloroquine—An optical coherence tomography angiography study. Graefe Arch Clin Exp Ophthalmol. 2020;258:2263–9. 10.1007/s00417-020-04788-4 PMC7550286 32533282

[24] Bao L, Zhou R, Wu Y, Wang J, Shen M, Lu F, Wang H, Chen Q. Unique changes in the retinal microvasculature reveal subclinical retinal impairment in patients with systemic lupus erythematosus. Microvasc Res. 2020;129:103957. 10.1016/j.mvr.2019.103957. Epub 2019 Nov 13. PMID: 31733303. 31733303 10.1016/j.mvr.2019.103957

[25] Lee HY, Chen J, Ying P, Xu SH, Kang M, Zou J, Liao XL, Shi W, Ling Q, Wang YX, et al. Investigation of altered retinal microvasculature in female patients with rheumatoid arthritis: optical coherence tomography angiography detection. Biosci Rep. 2023;43:BSR20230045. 37665319 10.1042/BSR20230045 PMC10578346

[26] Ayar K, Can ME, Koca N, Çelik D¸S. Evaluation of retinal vascularization by optical coherence tomography angiography (OCTA) in rheumatoid arthritis, and its relationship with disease activity. Mod Rheumatol. 2021;31:817–26. 32997565 10.1080/14397595.2020.1830740

[27] Iacono P, Da Pozzo S, Bedendo A, Arrigo A, Parravano M, Varano M, Battaglia Parodi M. OCT retinal angiography features in patients with rheumatoid arthritis: A pilot study. Eur J Ophthalmol. 2022;32(4):2433–9. Epub 2021 Jul 27. PMID: 34313159. 34313159 10.1177/11206721211035626

[28] Yener NP, Ayar K. Evaluation of retinal microvascular structures by optical coherence tomography angiography in primary Sjögren’s syndrome. Int Ophthalmol. 2022;42(4):1147–59. 10.1007/s10792-021-02100-9. Epub 2021 Nov 8. PMID: 34746971. 34746971 10.1007/s10792-021-02100-9

[29] Liu R, Wang Y, Li Q, Xia Q, Xu T, Han T, Cai S, Luo S, Wu R, Shao Y. Optical coherence tomography angiography biomarkers of retinal thickness and microvascular alterations in Sjogren’s syndrome. Front Neurol. 2022;13:853930. 10.3389/fneur.2022.853930. PMID: 35350402; PMCID: PMC8957855. 35350402 10.3389/fneur.2022.853930 PMC8957855

[30] Foti R, Zeppieri M, Foti R, Visalli E, Amato G, Amato R, Dammino E, D’Esposito F, Gagliano C. Retinal vascular abnormalities and clinical parameters in systemic sclerosis. J Clin Med. 2024;13(10):2738. 10.3390/jcm13102738. PMID: 38792282; PMCID: PMC11122651. 38792282 10.3390/jcm13102738 PMC11122651

[31] Ermurat S, Koyuncu K. Evaluation of subclinical retinal microvascular changes in systemic lupus erythematosus patients using optical coherence tomography angiography and its relationship with disease activity. Lupus. 2022;31(5):541–54. 35282713 10.1177/09612033221084222

[32] Shi W-Q, Han T, Liu R, Xia Q, Xu T, Wang Y, Cai S, Luo S-L, Shao Y, Wu R. Retinal microvasculature and conjunctival vessel alterations in patients with systemic lupus Erythematosus—An optical coherence tomography angiography study. Front Med. 2021;8:724283. 10.3389/fmed.2021.724283. [PMC free article][PubMed]. 10.3389/fmed.2021.724283 PMC8674305 34926488

[33] Jesus D, Rodrigues M, Matos A, Henriques C, Silva JAPD, Inês LS. Performance of SLEDAI-2K to detect a clinically meaningful change in SLE disease activity: A 36–month prospective cohort study of 334 patients. Lupus. 2019;28:607–12. 30895904 10.1177/0961203319836717

