# Supplementary material for: Assessment of retinal microvascular changes in patients with systemic lupus erythematosus using optical coherence tomography angiography
Source: Int J Retina Vitreous. 2025 May 8;11:55. doi: 10.1186/s40942-025-00677-2 (PMC12060311; doi:10.1186/s40942-025-00677-2)
Supplement: Supplementary file 1 — Supplementary Material 1 [file 40942_2025_677_MOESM1_ESM.docx]

# Fig. S1 (A, B)

0

10

20

30

40

50

60

Group 1

Group 2

Group 3

Superficial parafoveal VD %

**S**

**1**

**A**

53

53.5

54

54.5

55

55.5

56

56.5

57

57.5

58

Group 1

Group 2

Group 3

Deep parafoveal VD%

**S**

**1**

**B**

**Fig S1 A&B:**

There was a significantly lower superficial parafoveal vessel density in systemic lupus patients with nephritis (Group 1) and those without nephritis (Group 2) compared to healthy subjects (Group 3), with p1 = 0.060, p2 = 0.038, and p3 = 0.032.

Additionally, a significantly lower deep parafoveal vessel density was observed in Group 1 compared to Group 3 (p1 = 0.041), while the difference between Group 2 and Group 3 was not statistically significant (p2 = 0.232).

# Fig. S1C


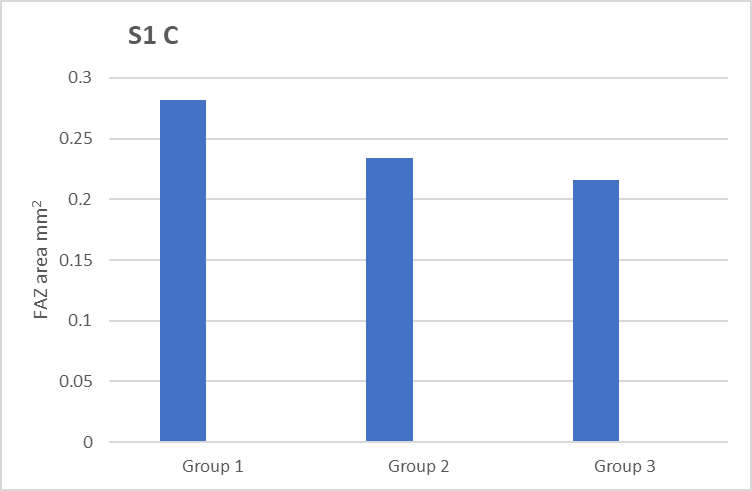


**Fig S1C:** There was an increase in the FAZ area in systemic lupus patients with nephritis (Group 1) and those without nephritis (Group 2) compared to healthy subjects (Group 3), with statistically significant differences (p1 = 0.029, p2 = 0.015).
